# Physical activity in patients with axial spondyloarthritis in a multi-ethnic south-east Asian country

**DOI:** 10.1186/s41927-021-00211-5

**Published:** 2021-08-31

**Authors:** Jie Kie Phang, Andrew Yu Keat Khor, Yu Heng Kwan, Chin Teck Ng, Warren Fong

**Affiliations:** 1 Department of Rheumatology and Immunology, Singapore General Hospital, Outram Rd, Singapore, 169608 Singapore; 2 Duke-NUS Medical School, 8 College Rd, Singapore, 169857 Singapore; 3 Department of Medicine, Yong Loo Lin School of Medicine, National University of Singapore, 10 Medical Dr, Singapore, 117597 Singapore

**Keywords:** Ankylosing spondylitis, Physical activity, Exercise

## Abstract

**Background:**

Patients with axial spondyloarthritis (axSpA) may experience spinal stiffness and pain, leading to reduced physical function and quality of life. Despite the benefits of physical activity (PA) and exercise, previous studies have demonstrated lower levels of PA among patients with axSpA. This study aims to examine the patterns of PA among patients with axSpA compared to the general population in a multi-ethnic Asian country.

**Methods:**

This was a cross-sectional study conducted between May 2016 and Jan 2017. Consecutive patients with axSpA were recruited at an outpatient rheumatology clinic at Singapore General Hospital, the largest tertiary hospital in Singapore. Controls were based on a previous cross-sectional study. PA was assessed using the Global Physical Activity Questionnaire (GPAQ).

**Results:**

Seventy-four patients with axSpA were recruited and compared with 2679 controls. Lower proportion of patients with axSpA met the WHO recommendations for PA (axSpA = 77.0%, controls = 89.7%, *p* <  0.001). More patients with axSpA had high level of sedentary activity compared to controls (axSpA = 56.8%, controls = 36.1%, *p* <  0.001). Levels of PA did not differ between patients with inactive versus active axSpA disease (*p* = 0.91).

**Conclusions:**

Proportion of patients with axSpA meeting the WHO recommendations for PA differed significantly from the general population, and level of PA did not differ between patients with active and inactive axSpA disease. Higher levels of sedentary activity were seen in patient with axSpA compared to the general population, highlighting the need for interventions to promote PA among patients with axSpA.

**Supplementary Information:**

The online version contains supplementary material available at 10.1186/s41927-021-00211-5.

## Introduction

Patients with axial spondyloarthritis (axSpA) have predominantly inflammatory back pain, and their axial skeleton and sacroiliac joints are often affected [1]. This leads to reduced spinal mobility and function [2], which may result in reduced physical functioning and quality of life (QOL) [3]. In addition, axSpA has also been shown to have increased risk of cardiovascular disease (CVD), when compared to the general population [4].

Physical activity (PA) and exercise have been shown to improve general well-being and reduce CVD risk in the general population [5]. PA is defined as ‘any bodily movement produced by skeletal muscles that result in energy expenditure’ [6]. A dose-response relationship between PA and health benefits has also been demonstrated [7]. The World Health Organisation (WHO) has set out recommendations for PA and muscle strengthening activities in adults, including the elderly and people with chronic conditions [8]. The benefits of PA in axSpA have also been shown [9], and the European League Against Rheumatism (EULAR) recommends PA as part of the non-pharmacological management in axSpA [10].

Despite these recommendations, studies show that levels of PA are reduced among patients with axSpA in Canada and Sweden [11, 12]. Based on our best knowledge, there is no study investigating the PA levels among patients with axSpA in Asia. The aim of this paper is to examine the patterns of PA among patients with axSpA compared to the general population in a multi-ethnic Asian country.

## Materials and methods

### Study design and population

This was a cross-sectional study conducted between May 2016 and Jan 2017. Consecutive patients with axSpA were recruited at an outpatient rheumatology clinic at Singapore General Hospital, a tertiary hospital and the largest hospital in Singapore. Inclusion criteria included clinical diagnosis of axSpA, at least 21 years old, and fulfilled the 2009 Assessment of Spondyloarthritis International Society (ASAS) Classification for axSpA classification criteria. Controls were based on a previous population-based cross-sectional Singapore Health 2012 study [13] where random households were selected from the Database of Dwellings, a comprehensive database of all residential dwelling units in Singapore, maintained by the Singapore Department of Statistics. Eligible households who consented to participate in the study received phone calls and household visits followed by an interview.

Demographics such as age, gender, race, body mass index (BMI), smoking status, alcohol status, employment, education level, total monthly household income and comorbidities (hypertension, diabetes mellitus, hyperlipidemia, coronary artery disease, cerebrovascular disease and chronic kidney disease) were collected via a standardised data collection form.

The study was approved by the Institutional Review Board (CIRB 2016/2210) and written informed consent was obtained from all participants.

### Assessment

#### Assessment of disease activity and function in patients with axSpA

Disease activity for axSpA was assessed using Ankylosing Spondylitis Disease Activity Score with C-Reactive Protein (ASDAS-CRP) [14], and function was assessed using the Bath Ankylosing Spondylitis Functional Index (BASFI) [15]. The ASDAS-CRP comprised of back pain, peripheral pain and swelling, duration of morning stiffness, patient global assessment using a 11-point numerical rating scale and the C-reactive protein. Inactive disease was defined as ASDAS < 1.3, low disease active as ASDAS ≥1.3 and ≤ 2.1, high disease activity as ASDAS > 2.1 and ≤ 3.5 and very high disease activity as ASDAS > 3.5(31). The BASFI is a validated index used to determine the degree of functional limitation. It is a self-assessment instrument comprising of 10 questions relating to function and patient’s ability to cope with everyday life, with questions answered on a 10 cm horizontal visual analog scale. Higher BASFI scores indicate greater functional limitation.

#### Assessment of physical activity

Physical activity was assessed using the Global Physical Activity Questionnaires (GPAQ) developed by the WHO [16]. Interviewers received training to administer the questionnaires to study participants. The intensity and frequency of moderate-to-vigorous PA (MVPA) was assessed under 3 domains- work, travelling and recreation. Physical activity data was processed and participants were defined as meeting or not meeting recommendations according the cut-offs in the GPAQ analysis guide [16]. We presented the median number of minutes spent on average per day, in work-, transport- and recreation-related physical activity. Participants who met the WHO recommendations on physical activity were those that achieved (i) 150 min of moderate-intensity physical activity or (ii) 75 min of vigorous-intensity physical activity or (iii) an equivalent combination of moderate- and vigorous-intensity physical activity achieving at least 600 MET-minutes per week. The level of PA was assessed according to the following criteria [17]:

High: A person reaching any of the following criteria is classified in this category. Vigorous intensity activity on at least three days achieving a minimum of 1500 MET-minutes per week or seven or more days of any combination of walking, moderate- or vigorous-intensity activities achieving a minimum of 3000 MET-minutes per week.

Moderate: A person not meeting the criteria for the ‘high’ category, but meeting any of the following criteria is classified in this category. Three or more days of vigorous-intensity activity of at least 20 min per day or five or more days of moderate intensity activity or walking for at least 30 min per day or five or more days of any combination of walking, moderate- or vigorous-intensity activities achieving a minimum of 600 MET-minutes per week.

Low: A person not meeting any of the abovementioned criteria falls in this category.

Sedentary behaviour was assessed using a single item in the GPAQ (How much time do you usually spend sitting or reclining on a typical day?). We defined sedentary as sitting for at least 8 h per day based on a previous study showing increased all-cause mortality with sitting for at least 8 or more hours per day [18].

### Statistical analysis

Data was tested for normality using Shapiro-Wilk test. Continuous variables were reported as means and standard deviation (SD) if normally distributed, or as median and interquartile range (IQR) if otherwise. Comparison of means were done using student t-test or one-way ANOVAs, or with non-parametric Mann-Whitney U-test or Kruskal-Wallis tests as appropriate. Comparison of medians were performed using nonparametric equality-of-medians test. Categorical variables were compared using chi-squared tests or Fisher exact test as appropriate. We also performed a sensitivity analysis for median number of minutes spent on average per day, in work-, transport- and recreation-related physical activity using sex and age matched controls. Statistical significance was set at *p* <  0.05 for all analyses. Analysis was performed using Stata version 15.

## Results

### Socio-demographic characteristics

Seventy-four patients with axSpA were recruited. They were compared to 2679 controls from the study by Win Am et al. [13]. The socio-demographic characteristics of the participants are presented in Table 1. Patients with axSpA were younger (median age [IQR], 37.0 [26.3] years) as compared to controls (median age [IQR], 51.0 [23.0] years) (*p* <  0.001). Majority of patients with axSpA were male (75.7%), which was higher than the proportion of males among the control group (44.8%) (*p* < 0.001). There was also a higher percentage of Chinese (87.8%) in the axSpA group as compared to controls (66.6%) (*p* < 0.001). BMI was similar between the 2 groups (axSpA: median BMI [IQR], 24.1 [5.1]; control: median BMI [IQR], 23.6 [5.5]; *p* = 0.29). All patients with axSpA were current smokers or ex-smokers (100%) while majority of controls did not smoke (70.7%) (*p* < 0.001). None of the participants in the control group had axial spondyloarthritis, whilst 58.8% self-reported a history of either osteoarthritis of the hip or knee, rheumatoid arthritis or gout.

**Table 1:** Characteristics and levels of activity of patients with axial spondyloarthritis versus controls

Characteristic	AxSpA *N* = 74	Controls *N* = 2679	*P* value
Current age, median (IQR), years	37.0 (26.3)	51.0 (23.0)	< 0.01
Sex, male, n (%)	56 (75.7)	1201 (44.8)	< 0.01
Race, Chinese, n (%)	65 (87.8)	1783 (66.6)	< 0.01
Body mass index, median (IQR), kg m^−2^	24.1 (5.1)	23.6 (5.5)	0.29
Smoking status			
Currently smoking, n (%)	26 (35.1)	335 (12.5)	< 0.01
Ex-smoker, n (%)	48 (64.9)	450 (16.8)	
Never smoked, n (%)	0 (0)	1894 (70.7)	
Alcohol drinkers, n (%)	48 (65.8)	1660 (62.0)	0.35
Education level			
Secondary and below, n (%)	74 (100)	1352 (50.5)	< 0.01
Diploma or equivalent, n (%)	0 (0)	674 (25.2)	
Degree, n (%)	0 (0)	653 (24.4)	
Employed, n (%)	57 (77.0)	1798 (67.1)	0.07
Total monthly household income*			
< SGD $2000, n (%)	12 (16.2)	633 (23.6)	0.23
SGD $2000–3999, n (%)	21 (28.4)	686 (25.6)	
SGD$4000–5999, n (%)	17 (23.0)	481 (18.0)	
≥ SGD$6000, n (%)	24 (32.4)	629 (23.5)	
Co-morbidities			
Hypertension, n (%)	11 (14.9)	499 (18.6)	0.41
Diabetes mellitus, n (%)	3 (4.1)	246 (9.2)	0.09
Hyperlipidemia, n (%)	6 (8.1)	681 (25.4)	< 0.01
Coronary artery disease, n (%)	4 (5.4)	105 (3.9)	0.33
Chronic kidney disease, n (%)	2 (2.7)	18 (0.67)	0.10
WHO recommendations on physical activity for health			
Meets WHO recommendations^†^, n (%)	57 (77.0)	2403 (89.7)	< 0.01
Median time of total physical activity on average per day (IQR), minutes	60 (107.1)	44.3 (82.1)	0.07
Median time spent on work-related physical activity on average per day (IQR), minutes	0 (31.0)	0 (34.3)	0.38
Median time spent on travel-related physical activity on average per day (IQR), minutes	13.6 (36.4)	20 (34.3)	0.07
Median time spent on recreational-related physical activity on average per day (IQR), minutes	13.9 (51.4)	0 (17.1)	0.01
Doing no work-, transport-, or recreation-related physical activity, n (%)	9 (12.2)	240 (9.0)	0.34
WHO classification of physical activity level			
Low level of physical activity, n (%)	17 (23.0)	1551 (57.9)	< 0.01
Moderate level of physical activity, n (%)	25 (33.8)	303 (11.3)	
High level of physical activity, n (%)	32 (43.2)	825 (30.8)	
High level of sedentary activity^§^, n (%)	42 (56.8)	966 (36.1)	< 0.01

Abbreviation

*AxSpA* Axial spondyloarthritis, *IQR* interquartile range, *SGD* Singapore dollar, *WHO* World Health Organization

*Some participants in the control group did not provide information on the household income.

†Meeting WHO recommendations on physical activity for health includes: (i) 150 min of moderate-intensity physical activity or (ii) 75 min of vigorous-intensity physical activity or (iii) An equivalent combination of moderate- and vigorous-intensity physical activity achieving at least 600 MET-minutes

§Defined as more than 8 h of sedentary activity

### Physical activity

Lower proportion of patients with AxSpA met the WHO recommendations for PA (AxSpA = 77.0%, controls = 89.7%, *p* < 0.001). In addition, a larger proportion of patients with axSpA were noted to have high levels of sedentary lifestyle compared to controls (56.8% vs 36.1% respectively, *p* < 0.001).

The median total amount of time spent on physical activity per day did not differ significantly between both groups (*p* = 0.07), but patients with axSpA spent more time on recreational- related physical activity per day (median [IQR], 13.9 [51.4] minutes vs 0 [17.1] minutes, *p* = 0.01) compared to controls. Results from the sensitivity analysis were largely similar (Supplementary Table 1).

### Patients with axSpA – inactive vs active disease

Demographics between patients with axSpA with inactive disease and those with active disease were not statistically different (Table 2). Comorbidities were similar between the inactive and active disease groups.

**Table 2:** Characteristics and physical activity levels between patients with axSpA with inactive vs low or high or very high disease activity

Characteristics	Inactive disease *N* = 27	Low, high or very high disease activity *N* = 47	*P* value
Current age, median (IQR), years	34.0 (23.0)	42.0 (28.0)	0.14
Sex, male, n (%)	19 (70.4)	37 (78.7)	0.42
Race, Chinese, n (%)	23 (85.2)	42 (89.4)	0.72
Body mass index, median (IQR), kg m^−2^	22.6 (5.2)	24.3 (4.9)	0.06
Smoking status			
Currently smoking, n (%)	8 (29.6)	18 (38.3)	0.45
Ex-smoker, n (%)	19 (70.4)	29 (61.7)	
Alcohol drinkers, n (%)	17 (63.0)	31 (67.4)	0.70
BASFI (range 0–10), median (IQR)	1.1 (0.6)	1.9 (2.0)	< 0.01
ASDAS-CRP (range 0–10), median (IQR)	1.1 (0.3)	2.0 (1.0)	< 0.01
Co-morbidities			
Hypertension, n (%)	4 (14.8)	7 (14.9)	1.00
Diabetes mellitus, n (%)	0 (0)	3 (6.4)	0.30
Hyperlipidemia, n (%)	2 (7.4)	4 (8.5)	1.00
Coronary artery disease, n (%)	1 (3.7)	3 (6.4)	1.00
Chronic kidney disease, n (%)	0 (0)	2 (4.3)	0.53
Employed, n (%)	22 (81.5)	35 (74.5)	0.49
WHO recommendations on physical activity for health			
Meets WHO recommendations^†^, n (%)	21 (77.8)	36 (76.6)	0.91
Median time of total physical activity on average per day (IQR), minutes	68.6 (145.7)	57.9 (85.7)	0.46
Median time spent on work-related physical activity on average per day (IQR), minutes	0 (34.3)	0 (17.1)	0.71
Median time spent on travel-related physical activity on average per day (IQR), minutes	8.6 (60.0)	14.3 (30.0)	0.65
Median time spent on recreational-related physical activity on average per day (IQR), minutes	12.9 (64.3)	15.0 (42.9)	0.37
Doing no work-, transport-, or recreation-related physical activity, n (%)	4 (14.8)	5 (10.6)	0.72
WHO level of physical activity			
Low level of physical activity, n (%)	6 (22.2)	11 (23.4)	0.47
Moderate level of physical activity, n (%)	7 (25.9)	18 (38.3)	
High level of physical activity, n (%)	14 (51.9)	18 (38.3)	
High level of sedentary activity^§^, n (%)	18 (66.7)	24 (51.1)	0.19

Abbreviation

*ASDAS-CRP* Ankylosing Spondylitis Disease Activity Score with C-Reactive Protein, *AxSpA* Axial spondyloarthritis, *BASFI* Bath Ankylosing Spondylitis Functional Index, *IQR* interquartile range, *WHO* World Health Organization

†Meeting WHO recommendations on physical activity for health includes: (i) 150 min of moderate-intensity physical activity or (ii) 75 min of vigorous-intensity physical activity or (iii) An equivalent combination of moderate- and vigorous-intensity physical activity achieving at least 600 MET-minutes

§Defined as more than 8 h of sedentary

Proportion of patients who met WHO recommendations for PA did not differ between patients with inactive versus active disease groups (77.8% vs 76.6%, *p* = 0.907), respectively). Patients with axSpA and inactive disease appeared to have higher high levels of PA compared to those with active disease (51.9% vs 38.3% respectively), though this difference was not statistically significant. Proportion of patients with axSpA and high levels of sedentary activity did not differ significantly between inactive and active disease (66.7% vs 51.1%, *p* = 0.19). Both groups spent more time on recreational activities per day compared to travelling and work.

## Discussion

This study examined the levels of PA among patients with axSpA in multi-ethnic Asian country, Singapore. To our knowledge, this is the first study in South-East Asia looking at PA in patients with axSpA. Our study found that patients with axSpA were less likely to meet WHO recommendations for physical activity but spent more time on recreational-related physical activities, as compared to controls. Patients with axSpA also had higher levels of sedentary behaviour compared to the general population.

In our study, a lower proportion of patients with AxSpA met the WHO recommendations for PA as compared to controls. The literature regarding PA in axial spondylitis has shown mixed results. When using accelometry to measure PA, Swinnen et al. reported lower PA levels in patients with axSpA compared to healthy matched controls [19], while Plasqui et al. reported no statistical difference in PA levels between patients with AS and healthy controls [20]. The cause of lower levels of self-reported physical activity in patients with axSpA could not be elucidated from our study, and further studies will be needed to identify the factors. Previous study had identified physical exertion as the main barrier for exercising among patients with axSpA [21].

The proportion of patients with axSpA who met WHO recommendations for PA in our study was high (77%) compared to other reported studies. A Swedish study by Haglund et al. using patient-reported physical activity level showed that 68% of patients with AS met WHO recommendations [22], compared to 65% in the general Swedish population. In a cross-sectional study in Paris based on self-reported physical activity level, 54.7% of the patients with axSpA met the WHO recommendations [21]. Interestingly, the levels of self-reported PA did not differ between patients with axSpA with inactive disease and patients with axSpA with active disease. A possible reason for this is although active disease has been shown to result in reduced physical activity [23], however, it is also possible that patients with more active disease may have been more motivated to exercise as demonstrated in the study by Falkenbach et al. [24], where patients with AS who were more disabled were found to exercise more frequently. Also, patients with axSpA with active disease might also have been encouraged by their rheumatologists to engage in physical activity since it is part of the numerous guidelines for the management of patients with axSpA with active disease [10, 25, 26].

The high level of sedentary behaviour noted in the axSpA group compared to controls is worrying as sedentary activity has been linked to increased cardiovascular risk and death [27], and it is known that patients with axSpA are at higher risk of cardiovascular disease [28]. Increase in physical activity has been shown to attenuate cardiovascular risk [29], and should be encouraged in patients with axSpA.

There are several limitations to this paper. Firstly, the small sample size prevented analysis among the different ethnic groups apart from comparison between Chinese and non-Chinese patients. Secondly, as this was a cross-sectional study, we were not able to determine whether patients had higher levels of PA before the onset of disease. Thirdly, smokers and patients with lower educational levels might have been over-represented in our study due to the small sample size, as the proportion of smokers and distribution of educational levels in this study differed from our previous cohorts [30, 31]. Repeating the study with larger sample size will be needed. Fourthly, while the GPAQ is a validated questionnaire [32], it is a self-reported questionnaire, and thus a subjective evaluation, which may have led to overestimation of PA [33]. Lastly, as the axSpA and control groups were different in terms of sociodemographic characteristics, there may be confounding in our data, which could have biased the estimate towards or away from the null. Older participants were more likely to meet PA recommendations [13], while smoking was associated with lower exercise levels [34]. An additional limitation of the study is that the population data for the controls was collected in 2012 while the data for patients with axSpA was collected in 2016. We could not rule out the possibility of changes in PA levels due to changes in the environment in the different time period.

## Conclusion

Although levels of PA did not differ between patients with active and inactive axSpA disease, patients with axSpA were less likely to meet the WHO recommendations for PA compared to the general population, and had higher levels of sedentary activity compared to the general population. This highlights the need for interventions to promote PA among patients with axSpA.

## Supplementary Information

**Additional file 1: Supplementary Table 1.** Levels of activity of patients with axial spondyloarthritis with age-, gender-matched controls.

## Data Availability

The datasets used and/or analysed during the current study are available from the corresponding author on reasonable request.
