# Supplementary material for: Physical activity in patients with axial spondyloarthritis in a multi-ethnic south-east Asian country
Source: BMC Rheumatol. 2021 Aug 31;5:38. doi: 10.1186/s41927-021-00211-5 (PMC8406584; doi:10.1186/s41927-021-00211-5)
Supplement: Supplementary file 1 — Additional file 1: Supplementary Table 1. Levels of activity of patients with axial spondyloarthritis with age-, gender-matched controls. [file 41927_2021_211_MOESM1_ESM.docx]

Supplementary table 1. Levels of activity of patients with axial spondyloarthritis with age-, gender-matched controls

| Characteristic | AxSpA  N=68 | Controls  N=68 | *P* value |
| --- | --- | --- | --- |
| Median time of total physical activity on average per day (IQR), minutes | 54.6 (101.8) | 44.3 (67.5) | 0.30 |
| Median time spent on work-related physical activity on average per day (IQR), minutes | 0 (32.1) | 0 (25.7) | 0.38 |
| Median time spent on travel-related physical activity on average per day (IQR), minutes | 11.1 (32.1) | 21.43 (38.9) | 0.17 |
| Median time spent on recreational-related physical activity on average per day (IQR), minutes | 12.9 (42.9) | 0 (15.0) | 0.003 |
